# Influence of perceptions of reporting nurses’ medical errors and patient safety culture on patient safety nursing practices in Korea: a cross-sectional study

**DOI:** 10.7586/jkbns.24.028

**Published:** 2025-02-25

**Authors:** Young Hee Kim, Mi Young Kim

**Affiliations:** 1 Graduate School of Clinical Nursing, Hanyang University, National Police Hospital, Depart of Deputy of Nursing, Seoul, Korea; 2 College of Nursing, Hanyang University, Seoul, Korea

**Keywords:** Perception, Patient safety, Nursing, Medical errors, Safety management

## Abstract

**Purpose:**

This study investigated how perceptions of reporting nurses’ medical errors and patient safety culture affected patient safety nursing practices.

**Methods:**

The participants in this descriptive study were 157 nurses at four medical institutions located in Seoul, South Korea, recruited by convenience sampling. A cross-sectional design was employed. The data were obtained through self-reporting questionnaires from August 13 to 28, 2021. Statistical analyses were performed using SPSS version 25.0. Descriptive statistics were obtained, and the t-test, analysis of variance, Scheffé test, Pearson correlation coefficient, and stepwise multiple regression were performed.

**Results:**

The factors influencing patient safety nursing practices were total practical experience as a nurse (≥ 6 years), perceptions of patient safety culture, and perceptions of reporting nurses’ medical errors. The overall explanatory power of these factors regarding patient safety nursing practices was 25.1%.

**Conclusion:**

To improve patient safety nursing practices, hospitals should provide education on patient safety nursing practices. Moreover, interventions should be developed and applied to promote the creation of a patient safety culture.

## INTRODUCTION

Patient safety is at the heart of public healthcare and an essential challenge in the relevant fields [1]. As a key determinant of the quality of healthcare services, patient safety is defined as the prevention of errors in providing healthcare services and the elimination or amelioration of patient injuries that can be caused by such errors [2]. Recently, awareness of the importance of patient safety has steadily increased. In particular, nurses at medical institutions take part in maintaining and increasing the level of patient safety at hospitals via nursing [3]. As a group of experts expected to sensitively perceive any problems of patient safety related to the prevention of falls, drug administration, and infection, nurses make a critical contribution to the improvement of patient safety [4,5].

A patient safety incident is an unforeseen event or accident that can cause patient harm [6]. The World Health Organization estimates that unsafe care affects millions of patients each year, with approximately 1 in 10 patients experiencing harm from adverse events while receiving healthcare [7]. To address this issue, member countries of the Organisation for Economic Co-operation and Development spend 15% of their total national hospital expenditure and activity on patient safety incidents [8]. The categories of patient safety incidents are near miss, adverse event, and sentinel event. Near miss is a case of an error that does not cause patient harm; adverse event is a case where an injury caused by a medical procedure demands treatment or intervention. Further, sentinel event is a case that causes unexpected death or loss of a major function in a patient [9]. According to a study analyzing the data of reports on patient safety for three years between 2018 and 2020, adverse events as a patient safety accident are influenced by the patient’s age, hospital bed number, level of medical institution, accident location, and accident type. Sentinel events are influenced by the patient’s sex and age, accident location, accident type, and nurses’ work hours [10]. For the type of patient safety accidents, the percentage of fall accidents is the highest in all three categories of near miss, adverse event, and sentinel event. Compared with infection and contamination accidents, fall accidents increase the risk of sentinel event by 2.7 times [10]. According to the Korea Patient Safety Reporting & Learning System, the number of reported incidents was 11,935 in 2019, 13,919 in 2020, 13,416 in 2021, 14,820 in 2022, and 1,779 as of January 2023, with ≥ 1,000 cases per month since April 2022 [11]. The data are from voluntary reports by medical institutions; as such, the actual number of incidents may be higher [12].

Relevant measures are essential to reduce patient safety incidents and improve overall patient safety. To advance healthcare quality and patient safety, South Korea has operated a medical institution accreditation system since 2004 [13]. This system, through regular preparation and education on assessment items conducted every four years, has significantly contributed to enhancing the quality of clinical nursing services. Notably, it has improved the perception of patient examination as a key measure to prevent safety incidents [14]. However, despite these efforts to bolster patient safety and elevate the quality of clinical nursing services, the extent of improvement in nurses' perceptions achieved through this system has not been thoroughly investigated.

Meanwhile, patient safety incidents are associated with medical errors managed after their reporting. The reporting of nurses’ medical errors is the reporting of all potential errors, mistakes, and accidents in the healthcare delivery system regardless of patient harm [15]. Perception of medical error reporting refers to the attitudes, beliefs, concerns, and level of knowledge related to reporting such medical errors among healthcare professionals [16]. Through such reports, staff nurses can share information about the incident with the senior nurse and medical institution. Healthcare professionals must empathize with the need to report nurses’ medical errors toward enhanced patient safety [17,18]. Furthermore, to reduce the rate of medical errors, medical institutions must emphasize the improvement of the patient safety culture and the establishment of a non-punitive system for reporting nurses’ medical errors [17]. Nonetheless, as cases of non-reporting of medical errors remain owing to the organizational culture [19], studies should be conducted on the change in the level of perception in nurses regarding the reporting of errors.

The perception of patient safety culture reflects the structure of beliefs, values, and behaviors shared at the individual, department, and organization levels to minimize and prevent medical errors that can arise in a healthcare setting [16]. Perception of patient safety culture refers to the shared awareness among organizational members regarding the importance of patient safety [15]. The patient safety practices by a team of healthcare professionals at a medical institution could vary according to how patient safety culture is perceived—the perception of patient safety culture reflects system-related factors, including the measures taken in the event of a patient safety incident. For a patient safety incident, the patient’s guardian would question what caused the incident, whereas the healthcare staff would experience the fear of a potential lawsuit, deteriorated relationship with the patient, and a damaged reputation as a professional [1]. Indeed, the perception of patient safety culture has been shown to affect patient safety nursing practices by nurses at general hospitals [20]. With recent changes focusing on patient safety across medical institutions, scholars must investigate the perception of nurses, who are in the closest contact with patients, regarding patient safety and how their perception affects patient safety nursing practices.

The increased specialization in and division of nursing tasks have increased nurses’ workload [21]. To further emphasize nurses’ role in patient safety nursing practices, in-depth analyses and investigations on patient safety culture and patient safety nursing practices are necessary. Moreover, with the emphasis on the perceptions of reporting nurses’ medical errors and patient safety culture, their respective levels should be determined. Thus, this study aimed to investigate the effects of the perceptions of reporting nurses’ medical errors and patient safety culture on patient safety nursing practices.

This study aimed to determine the effects of the nurses’ perceptions of reporting nurses’ medical errors and patient safety culture on their patient safety nursing practices. The specific objectives are as follows:

1) Analyze the differences in nurses’ perceptions of reporting nurses’ medical errors and patient safety culture and nursing practices according to general characteristics;

2) Analyze the correlations across the perceptions of reporting nurses’ medical errors and patient safety culture and nursing practices;

3) Determine the influencing factors of patient safety nursing practices.

## METHODS

### 1. Study design

This study is a descriptive research study to explore the impact of nurses' awareness of medical error reporting and patient safety culture on safe nursing activities.

### 2. Participants

The participants were nurses currently employed at four medical institutions (National Police Hospital, Kangdong Sacred Heart Hospital, Seoul Sacred Heart General Hospital, and Wooridul Hospital Seoul Airport Gimpo) with 200–600 beds (one university hospital, one national general hospital, and two general hospitals) in Seoul, South Korea. Our sample included 157 participants.

To estimate the sample size, we used G*Power 3.1.9.7 [22] based on Cohen’s d calculations. The conditions for the multiple regression analysis included a medium effect size of .15, which was based on the general rules or norms described in the literature when specific prior information is lacking due to insufficient evidence from previous studies to derive an appropriate effect size [23]. Additionally, a significance level of .05 and a statistical power of .90 were set, with 11 predictors: sex, age, marital status, educational level, position, religion, total practical experience as a nurse, number of patients, patient safety education experience, perception of reporting nurses’ medical errors, and perception of patient safety culture. The estimated sample size was n = 152. Considering a dropout rate of approximately 20%, we distributed 182 questionnaires and retrieved 165 (91.6%). After removing eight questionnaires with insincere responses, we analyzed data from 157 questionnaires.

For eligibility, nurses with ≥ 6 months of practical experience of patient care were included in reference to a previous study reporting that a minimum of six months is necessary for a nurse to perform the tasks related to clinical nursing independently [21]. The exclusion criteria were as follows: nurses working in departments unrelated to direct patient care, insurance review, purchase, distribution, and administration and those working in health examination centers.

### 3. Measures

#### 1) Perception of reporting nurses’ medical errors

Perception of reporting nurses’ medical errors refers to the series of steps in informing a colleague, a senior nurse, or the medical institution of any forms of errors, mistakes, or accidents during nursing care in the process of healthcare service whereby patient harm has been caused, regardless of the outcome [15]. To measure the perception of reporting nurses’ medical errors, we used the tool developed [16]. The instrument comprises 17 questions regarding concerns on how the report would be used in evaluations, the belief that the report would lead to positive effects, the intention to report, and the level of knowledge on the reporting, as well as other factors in the context of reporting an event. Each question is rated on a 5-point Likert scale; higher scores indicate a more positive perception of reporting nurses’ medical errors. The reliability of the tool was Cronbach’s α = .80 at the time of tool development [16] and Cronbach’s α = .75 in our study.

#### 2) Perception of patient safety culture

Perception of patient safety culture reflects how the importance of patient safety is perceived commonly by every member of the healthcare organization [15]. To measure the perception of patient safety culture, we used the Hospital Survey on Patient Safety Culture developed by Agency for Healthcare Research and Quality [15] and subsequently translated, modified, and validated [24]. The tool comprises six subcategories with 44 questions, addressing the following: perception of the patient safety culture within the department; attitudes of the direct superiors and managers; perception of the reporting system for medical accidents; frequency of reporting of accidents; perception of patient safety culture within the hospital; general safety level in the department; frequency of reporting of errors in the past one year. Each question is rated on a 5-point Likert scale; higher scores indicate a more positive perception of the patient safety culture. The reliability of the tool was Cronbach’s α = .90 [24] and Cronbach’s α = .91 in our study.

#### 3) Patient safety nursing practices

Patient safety nursing practices refer to practices aimed at preventing patient injuries or accidents that could occur during the delivery of healthcare service [15]. To measure the patient safety nursing practices, we used the tool developed by the Korea Institute for Healthcare Accreditation and validated through revision and modification [25]. The research instrument for safety nursing activities consists of a total of 37 items, categorized as follows: five items on accurate patient identification, seven items on communication, three items on marking surgical or procedural sites, six items on fall prevention activities, four items on hand hygiene, two items on high-risk medication management, two items on facility environment management, four items on fire prevention and smoking cessation, and four items on inspection of medical equipment. The items are rated on a 5-point Likert scale, with higher scores indicating higher levels of patient safety nursing practices. The reliability of the tool was Cronbach’s α = .98 [25] and Cronbach’s α = .97 in our study.

### 4. Data collection

The data were collected from August 13 to 28, 2021. Using convenience sampling, we recruited participants after explaining the study purpose and goals to the department of nursing of the four medical institutions. The researcher visited the nursing department, explained the purpose and method of the study, and obtained consent for participation before conducting the survey. Before the survey, participants were informed that the collected data would never be used for purposes other than the study. Detailed explanations were provided about the confidentiality and anonymity of the survey. Participants were assured that their participation was entirely voluntary, and they could withdraw from the survey at any time without any disadvantages. The survey responses were collected anonymously and stored in a secure, locked storage box. Each piece of collected data was assigned a serial number for management. To minimize the influence of department heads, participants wrote their responses on an explanation sheet, sealed them in an envelope, and deposited them into a secure collection box placed in the department. For other hospitals, approximately 35 questionnaires were distributed to three hospitals through hospital acquaintances. Respondents filled out the survey, placed it in an envelope, and returned it via mail.

### 5. Data analysis

We used IBM SPSS version 25.0 Statistics for Windows (IBM., Armonk, NY, USA). We obtained the mean, standard deviation, frequency, and percentage values for nurses’ general characteristics, perception of reporting nurses’ medical errors, perception of patient safety culture, and patient safety nursing practices. To analyze the differences in these variables according to nurses’ general characteristics, we conducted an independent *t*-test and ANOVA, using the Scheffé test as the post-hoc test. Further, we used Pearson correlation coefficients to analyze the correlations across the variables. Additionally, we performed stepwise multiple linear regression analysis to identify the influencing factors of patient safety nursing practices. Statistical significance was set at *p* < .05.

### 6. Ethical considerations

This study was conducted with the approval of the institutional review board of National Police Hospital (IRB-202107-HR-008). We explained the purpose and methods of our study to the participants, and only those who voluntarily agreed to participate were recruited. Prior to the study, the participants were informed about privacy protection and anonymity and that the collected data would be used solely for research purposes. We highlighted that their participation should be voluntary and that they may withdraw at any time without any disadvantage. The participants were guided to check “voluntary participation” on the questionnaire before completing it. All questionnaires were anonymized. The data are to be stored for three years from the date of the completion in a locked storage cabinet.

## RESULTS

### 1. Participants’ general characteristics

The general characteristics of the nurses are as shown in Table 1. There were 148 women (94.3%), with 61 individuals (38.9%) aged 31~40 years. A majority of 97 participants (61.8%) were single, and 91 participants (58.0%) reported having no religion. Regarding education, 106 participants (67.5%) held a bachelor's degree as their highest level of education, and 136 participants (86.6%) were staff nurses. 41 (26.1%) had 1~4.9 years of clinical experience, 90 (57.3%) worked in the ward, 116 (73.9%) worked in three shifts, 58 (36.9%) were in charge of five or fewer patients, 50 (31.8%) had experience in patient safety education once a quarter, and 92 (58.6%) worked an average of 40~49 hours per week.

### 2. The levels of the participants’ perception of reporting nurses’ medical errors, perception of perception of patient safety culture, and patient safety nursing practices

Table 2 presents the levels of the participants’ perception of reporting nurses’ medical errors, perception of patient safety culture, and patient safety nursing practices for each investigated item. The average score for patient safety culture was 3.38 ± 0.37 on a 5-point scale. Among the subdomains, the attitude of immediate supervisors and managers scored the highest at 3.77 ± 0.49, followed by the perception of the medical error reporting system (3.54 ± 0.53), the evaluation of patient safety within the department (3.38 ± 0.68), the perception of patient safety culture within the department (3.31 ± 0.40), the ﻿frequency of accident reporting (3.28 ± 0.70), and ﻿awareness of patient safety culture in the hospital (3.28 ± 0.54). The average score for nurses' safety nursing activities was 4.07 ± 0.52 on a 5-point scale. Among the subdomains, hand hygiene scored the highest at 4.31 ± 0.57, followed by high-risk medication management (4.14 ± 0.65), accurate patient identification (4.12 ± 0.54), surgical/procedural site marking (4.12 ± 0.59), fall prevention activities (4.12 ± 0.59), fire prevention and smoking cessation (4.09 ± 0.66), medical device inspection (3.97 ± 0.70), communication (3.91 ± 0.57), and facility/environment inspection (3.81 ± 0.84).

### 3. Differences in awareness of medical error reporting, patient safety culture awareness, and patient safe nursing activities according to general characteristics

Table 3 presents the variations in the perception of reporting nurses’ medical errors, perception of patient safety culture, and patient safety nursing practices according to general characteristics. Notably, we found no significant differences in patient safety nursing practices according to general characteristics. Significant differences were observed based on religion (t = –2.74, *p* = .007), position (t = –2.44, *p* = .017), the number of patients under care (F = 3.94, *p* = .005), and average weekly working hours (F = 10.42, *p* < .001). Participants with no religious affiliation and charge nurses, compared to staff nurses, showed significantly higher perceptions of patient safety culture, especially among those who had not experienced medical errors.

### 4. Correlation between medical error reporting awareness, patient safety culture awareness, and safe nursing activities

Table 4 presents the correlations among the perception of reporting nurses’ medical errors, perception of patient safety culture, and patient safety nursing practices. Patient safety nursing practices showed a positive (+) correlation with the perception of reporting nurses’ medical errors (r = .22, *p* = .005) and patient safety culture (r = .24, *p* = .002). The positive (+) correlation between the perception of reporting nurses’ medical errors and that of patient safety culture was statistically significant (r = .35, *p* < .001).

### 5. Influence on nurses’ safety nursing activities

Table 5 presents the results of the multiple regression analysis conducted to identify the influencing factors of patient safety nursing practices. For the regression analysis on perceptions of patient safety nursing practices, variables that could significantly impact the outcome variable were included. Even if no statistically significant differences were observed in the univariate analysis of this study, variables deemed meaningful based on prior research were incorporated. The dependent variable was the perception of patient safety nursing practices, while the independent variables in the stepwise regression included educational level, position, total years of practical nursing experience, number of patients, perception of reporting nurses’ medical errors, and perception of patient safety culture. Additionally, when including variables in the analysis, they were reclassified into dichotomous categories based on clinical significance, and categorical variables were processed as dummy variables for inclusion. In this study, the division based on six years of experience was referenced from prior research [26], which developed and validated a clinical career management system for nurses in Korea. According to this system, the categories of Novice, Advanced Beginner, and Competent correspond to nurses with less than 5~6 years of experience, while Proficient and Expert refer to those with six or more years of experience.We checked the tolerance and variance inflation factor (VIF) to test the multicollinearity of independent variables. The VIF was 1.09~1.56, well within the threshold of 10.0. The Durbin–Watson statistics (2.03) also confirmed the lack of auto-correlation, whereas the tolerance (0.64~0.92) was below the threshold of 1.0, indicating the lack of multicollinearity. The multiple regression analysis results revealed that the factors that influenced patient safety nursing practices were total practical experience as a nurse (β = .31, *p* = .027), perception of patient safety culture (β = .30, *p* = .030), and perception of reporting nurses’ medical errors (β = .30, *p* = .035). The overall explanatory power of these factors on patient safety nursing practices was 25.1%.

## DISCUSSION

We investigated the effects of the nurses’ perceptions of reporting nurses’ medical errors and patient safety culture on patient safety nursing practices to provide basic data for developing plans to improve patient safety nursing practices. The level of patient safety nursing practices was higher in nurses with total practical experience as a nurse of ≥ 6 years, more positive perception of reporting nurses’ medical errors, and more positive perception of patient safety culture; the explanatory power of these factors on patient safety nursing practices was 25.1%. Our finding that total clinical experience as a nurse influences patient safety nursing practices partially supports previous reports indicating that nurses in higher positions score higher on patient safety nursing practices [27]. However, since the influence of job position on patient safety nursing practices was not significant in this study, it is interpreted as being attributable to the accumulation of experience rather than the position itself. Nevertheless, caution is required in interpreting these results, and further research on this topic is needed. Regarding patient safety nursing practices, nursing departments should regularly review and implement corrective and preventive measures to address adverse injuries or potential safety issues in nursing practices [28]. Hence, nurses with a level of experience that allows higher competence in basic tasks are likely to monitor and improve any factors related to patient safety, compared with nurses with little experience, which could presumably explain our results. Moreover, experienced nurses and nurse managers are predicted to lead in creating changes in the safety-related organizational culture and adopt the respective roles in actual patient safety nursing practices.

Meanwhile, a more positive perception of reporting nurses’ medical errors was correlated with a higher level of patient safety nursing practices. This coincided with a previous finding that self- reporting in the reporting system for medical accidents that may lead to safety accidents increases as the knowledge and information on medical errors decrease, and the positive perception of reporting medical errors decreases with anxiety over punitive or judgmental repercussions [29]. In other words, the level of patient safety nursing practices would increase when the nurses perceive that patient safety could be improved through the reporting of medical errors rather than when negative aspects, such as punitive actions, are emphasized. Strategies to enhance patient safety culture may include fostering a transparent reporting culture, implementing standardized processes, improving teamwork and communication, and conducting continuous monitoring and evaluation.

Among our participants, the perception of reporting nurses’ medical errors scored 3.35 ± 0.37 (out of 5), indicating a moderate level. By subcategory, the intention to report the incident scored the highest at 3.98 ± 0.64 (out of 5), which lent support to a study on the reporting and disclosure of nurses’ medical errors at tertiary hospitals—nurses tended to perceive the need for reporting medical errors [30]. However, public healthcare organizations in South Korea are known to have a culture of silence and criticism and a tendency to cover up patient safety incidents [31]. Thus, although the nurses may have moderate levels of perception and knowledge on the need to report errors, emotional support should be provided for them to report medical errors. Additionally, a non-punitive culture must be established for the reporting of errors in practice.

Another influencing factor of patient safety nursing practices was the perception of patient safety culture. Similarly, Kwon [25] reported a correlation between the perception of patient safety culture and the patient safety nursing practices and where the increased perception of patient safety culture led to the increased patient safety nursing practice. Thus, nurses with a more positive perception of the patient safety culture would more actively comply with patient safety nursing practices. In a study on self-reporting in the reporting system for medical incidents that could lead to safety accidents, Moon and Yun [29] found that reporting rates were lower than the actual occurrence of events. This was attributed to insufficient knowledge and information about medical errors, concerns about punitive or judgmental repercussions, and a lack of awareness about the importance of reporting. This, in line with our study, suggests that the perception of patient safety culture affects patient safety nursing practices.

Our analysis of the differences in the perception of reporting nurses’ medical errors showed that the perception was higher in women nurses than in men and charge nurses than staff nurses. Considering that the attitudes on the reporting of patient safety accidents in previous studies are of higher levels in women nurses and chief nurses [30], the level of perception may be higher in nurses in charge and chief nurses who view patient safety from a systemwide perspective rather than as individual cases. We also found that nurses with 6–10 patients under their care reported a more positive perception of the patient safety culture compared with nurses caring for a greater number of patients. This finding aligned with a previous finding of nurses with a higher workload having lower levels of perception on patient safety culture and performance of nursing tasks [24]. Thus, patient safety may be jeopardized by challenges in patient safety nursing practices unless the problems of insufficient personnel and nurse allocation are improved. However, since nurses caring for fewer than five patients did not exhibit higher perceptions of patient safety culture, it is challenging to interpret this solely based on the number of nurses or workload. Therefore, caution is needed in interpreting these findings, and further research is required.

Additionally, there have been cases where medical errors are not reported owing to the lack of efforts to improve the punitive and judgmental culture at medical institutions regarding the reporting of nurses’ medical errors [18]. Thus, hospitals’ organizational culture should be improved to move away from punitive and judgmental actions against the reporting of nurses’ medical errors. The departments and organizations must endeavor to prevent the recurrence of errors by collecting feedback on medical errors, thereby enhancing patient safety nursing practices.

## CONCLUSION

This study explored the effects of nurses’ perceptions of reporting nurses’ medical errors and perception of the patient safety culture on patient safety nursing practices. The results showed that nurses’ total practical experience as a nurse, perception of patient safety culture, and perception of reporting nurses’ medical errors were the influencing factors of patient safety nursing practices. The results will serve as basic data for implementing safety education and monitoring nurses with a low level of total practical nursing experience, which may help improve patient safety nursing practices. Moreover, our findings can benefit the development of various safety education programs aimed at patient safety nursing practices that incorporate the perceptions of reporting nurses’ medical errors and patient safety culture as key variables.

Furthermore, the significance of this study lies in providing a foundation for utilizing indicators commonly used in basic nursing, such as patient safety adverse events and medication errors, when addressing patient safety issues in the future. The study's findings have limitations in terms of generalizability due to the restricted sample and setting. Additionally, the researchers selected a medium effect size while considering the specific context of the study and the practical significance of the effect size, which is another limitation that should be taken into account when interpreting the results. However, this study provides valuable insights, suggesting the inclusion of basic nursing indicators as part of the factors influencing patient safety nursing in future research.

## Figures and Tables

**Table 1.** Occupational and General Characteristics of Participants (N = 157)

Characteristics	Categories	n (%)
Sex	Women	148 (94.3)
	Men	9 (5.7)
Age (yr)	20~30	57 (36.3)
	31~40	61 (38.9)
	41~50	31 (19.7)
	≥ 51	8 (5.1)
Marital status	Single	97 (61.8)
	Married	60 (38.2)
Religion	Yes	66 (42.0)
	No	91 (58.0)
Educational level	Undergraduate	37 (23.6)
	Bachelor’s degree	106 (67.5)
	Master’s degree or higher	14 (8.9)
Position	Staff nurse	136 (86.6)
	Charge nurse	21 (13.4)
Total practical experience as a nurse (yr)	＜1	6 (3.8)
	1~4.9	41 (26.1)
	5~9.9	41 (26.1)
	10~14.9	34 (21.7)
	15~19.9	24 (15.3)
	≥ 20	11 (7.0)
Work department	Ward	90 (57.3)
	Intensive care unit	11 (7.0)
	Emergency room	18 (11.5)
	Operating room	30 (19.1)
	Dialysis room	8 (5.1)
Work type	Three-shift work	116 (73.9)
	Fixed work	41 (26.1)
Number of patients	< 5	58 (36.9)
	6~10	32 (20.4)
	11~15	39 (24.8)
	16~20	13 (8.3)
	≥ 21	15 (9.6)
Average working hours per week	＜ 40	46 (29.3)
	40~49	92 (58.6)
	50~59	19 (12.1)
Patient safety education experience	Once a week	6 (3.8)
	Once a month	39 (24.8)
	Quarterly	50 (31.8)
	Semi-annual, once	25 (15.9)
	Once a year	37 (23.7)

**Table 2.** Levels of Perceptions of Reporting Nurses’ Medical Errors, Perceptions of Patient Safety Culture, and Patient Safety Nursing Practices (N = 157)

Categories		M ± SD
Perceptions of reporting nurses’ medical errors	Concerns on the use of evaluation	2.58 ± 0.66
	Belief in the improvement effect	3.83 ± 0.63
	Intention to report the incident	3.98 ± 0.64
	Knowledge of incident reporting	3.56 ± 0.77
	Total (out of 5)	3.35 ± 0.37
Perceptions of patient safety culture	Recognition of patient safety culture in the department	3.31 ± 0.40
	Attitudes of direct superiors and managers	3.77 ± 0.49
	Awareness of the medical accident reporting system	3.54 ± 0.53
	Frequency of accident reporting	3.28 ± 0.70
	Evaluation of patient safety in the department	3.38 ± 0.68
	Awareness of patient safety culture in the hospital	3.28 ± 0.54
	Total (out of 5)	3.38 ± 0.37
Patient safety nursing practices	Patient identification	4.12 ± 0.54
	Communication	3.91 ± 0.57
	Surgery/procedure	4.12 ± 0.59
	Fall prevention activities	4.12 ± 0.59
	Hand hygiene	4.31 ± 0.57
	High-risk drugs	4.14 ± 0.65
	Fire and no smoking	4.09 ± 0.66
	Facility/environment	3.81 ± 0.84
	Medical equipment	3.97 ± 0.70
	Total (out of 5)	4.07 ± 0.52

M = Mean; SD = Standard deviation.

**Table 3.** Differences in Awareness of Medical Error Reporting, Patient Safety Culture Awareness, and Patient Safe Nursing Activities According to General Characteristics (N = 157)

Characteristics	Categories	Perceptions of reporting nurses’ medical errors		Perceptions of patient safety culture		Patient safety nursing practices	
		M ± SD	t/F (*p*)	M ± SD	t/F (*p*)	M ± SD	t/F (*p*)
Sex	Women	3.36 ± 0.38	2.45 (.027)	3.38 ± 0.37	0.27 (.785)	4.08 ± 0.51	1.70 (.090)
	Men	3.22 ± 0.15		3.34 ± 0.47		3.78 ± 0.47	
Age (yr)	20~30	3.34 ± 0.34	0.89 (.449)	3.45 ± 0.38	1.43 (.236)	4.09 ± 0.51	1.34 (.263)
	31~40	3.31 ± 0.38		3.31 ± 0.38		4.00 ± 0.51	
	41~50	3.42 ± 0.37		3.36 ± 0.30		4.06 ± 0.49	
	≥ 51	3.49 ± 0.56		3.43 ± 0.47		4.38 ± 0.61	
Marital Status	Single	3.33 ± 0.35	−0.84 (.404)	3.38 ± 0.37	0.35 (.730)	4.02 ± 0.52	−1.35 (.179)
	Married	3.39 ± 0.41		3.36 ± 0.38		4.14 ± 0.51	
Religion	Yes	3.32 ± 0.43	−1.02 (.308)	3.28 ± 0.33	−2.74 (.007)	4.03 ± 0.52	−0.81 (.421)
	No	3.38 ± 0.32		3.44 ± 0.39		4.09 ± 0.52	
Educational level	Undergraduate	3.45 ± 0.37	1.69 (.189)	3.40 ± 0.35	1.20 (.306)	3.96 ± 0.51	1.09 (.339)
	Bachelor’s degree	3.33 ± 0.36		3.39 ± 0.38		4.09 ± 0.53	
	Master’s degree or higher	3.29 ± 0.45		3.23 ± 0.36		4.15 ± 0.42	
Position	Staff nurse	3.33 ± 0.36	−2.32 (.022)	3.36 ± 0.39	−2.44 (.017)	4.08 ± 0.53	0.92 (.361)
	Charge nurse	3.53 ± 0.39		3.48 ± 0.16		3.97 ± 0.42	
Total practical experience as a nurse (yr)	< 1	3.26 ± 0.33	1.70 (.139)	3.54 ± 0.61	0.48 (.791)	4.30 ± 0.56	1.47 (.202)
	1~4.9	3.30 ± 0.37		3.40 ± 0.36		4.01 ± 0.55	
	5~9.9	3.31 ± 0.36		3.33 ± 0.43		3.95 ± 0.50	
	10~14.9	3.32 ± 0.30		3.35 ± 0.28		4.10 ± 0.49	
	15~19.9	3.54 ± 0.42		3.41 ± 0.31		4.13 ± 0.49	
	≥ 20	3.43 ± 0.47		3.36 ± 0.40		4.33 ± 0.50	
Work department	Ward	3.38 ± 0.36	0.53 (.714)	3.38 ± 0.39	0.45 (.774)	4.04 ± 0.53	0.78 (.541)
	Intensive care unit	3.38 ± 0.42		3.33 ± 0.41		4.05 ± 0.40	
	Emergency room	3.29 ± 0.38		3.35 ± 0.42		4.26 ± 0.55	
	Operating room	3.33 ± 0.42		3.43 ± 0.26		4.06 ± 0.46	
	Dialysis room	3.23 ± 0.22		3.24 ± 0.32		3.98 ± 0.60	
Work type	3-shift work	3.38 ± 0.36	1.34 (.182)	3.38 ± 0.39	−0.05 (.961)	4.07 ± 0.52	0.18 (.859)
	Fixed work	3.29 ± 0.40		3.38 ± 0.31		4.05 ± 0.50	
Number of patients	< 5^a^	3.31 ± 0.37	1.78 (.136)	3.39 ± 0.34	3.94 (.005)	4.08 ± 0.51	0.18 (.947)
	6~10^b^	3.34 ± 0.36		3.54 ± 0.39	b > e^†^	4.07 ± 0.52	
	11~15^c^	3.49 ± 0.38		3.33 ± 0.34		4.04 ± 0.48	
	16~20^d^	3.30 ± 0.31		3.34 ± 0.32		4.16 ± 0.61	
	≥ 21^e^	3.27 ± 0.38		3.11 ± 0.40		4.00 ± 0.57	
Average working hours per week	< 40^a^	3.38 ± 0.35	0.17 (.847)	3.56 ± 0.30	10.42 (< .001)	4.00 ± 0.50	1.43 (.243)
	40~49^b^	3.34 ± 0.38		3.33 ± 0.38	a > b,c^†^	4.12 ± 0.52	
	50~59^c^	3.37 ± 0.41		3.17 ± 0.33		3.95 ± 0.52	
Patient safety education experience	Once a week	3.28 ± 0.36	0.78 (.542)	3.25 ± 0.24	0.96 (.433)	3.25 ± 0.24	0.96 (.433)
	Once a month	3.34 ± 0.34		3.46 ± 0.33		3.46 ± 0.33	
	Quarterly	3.43 ± 0.34		3.37 ± 0.41		3.37 ± 0.41	
	Semi-annual, once	3.33 ± 0.44		3.38 ± 0.42		3.38 ± 0.42	
	Once a year	3.30 ± 0.40		3.31 ± 0.33		3.31 ± 0.33	

Different superscripts indicate significant differences between groups (*p* < 0.05). M = Mean; SD = Standard deviation. ^†^Scheffe test.

**Table 4.** Correlations between Perceptions of Reporting Nurses’ Medical, Perceptions of Patient Safety Culture, and Patient Safety Nursing Practices (N = 157)

Variables	Perceptions of reporting nurses’ medical errors	Perceptions of patient safety culture	Patient safety nursing practices
	r(*p*)	r(*p*)	r(*p*)
Perceptions of patient safety culture	.35 (< .001)	1	-
Patient safety nursing practices	.22 (.005)	.24 (.002)	1

**Table 5.** Factors that Influence Nurses’ Patient Safety Nursing Practices (N = 157)

Variables	B	SE	β	t	*p*	VIF
(Constant)	0.77	0.82		0.94	.354	
Education level (ref. undergraduate)	0.13	0.18	.10	0.72	.477	1.33
Position (ref. staff nurse)	−0.30	0.22	−.21	−1.37	.177	1.56
Total practical experience as a nurse (yr) (ref. 5 years or less)	0.33	0.14	.31	2.29	.027	1.34
Number of patients (ref. 5 or fewer)	0.03	0.22	.01	0.12	.908	1.09
Perception of reporting nurses’ medical errors	0.43	0.12		2.18	.035	1.33
Perception of patient safety culture	0.49	0.22	.30	2.23	.030	1.30
R² = .33, adj R² = .25, F = 4.02 (*p* < .001), Durbin–Watson = 2.03						

Dummy variable: Final degree (≥ bachelor’s degree: 1), position (charge nurse: 1), total practical experience as a nurse (≥ 6 years: 1), number of patients (≥ 6: 1). SE = Standard error; VIF = Variance inflation factor; ref = reference.

## Data Availability

Please contact the corresponding author for data availability.
